# Environmental and Sex Effects on Bacterial Carriage by Adult House Flies (*Musca domestica* L.)

**DOI:** 10.3390/insects11070401

**Published:** 2020-06-28

**Authors:** Saraswoti Neupane, Kotie White, Jessica L. Thomson, Ludek Zurek, Dana Nayduch

**Affiliations:** 1 Department of Entomology, Kansas State University, Manhattan, KS 66506, USA; sneupane@ksu.edu (S.N.); jessiel@ksu.edu (J.L.T.); 2 College of Veterinary Medicine, Kansas State University, Manhattan, KS 66506, USA; kotielanewootten@gmail.com; 3 Central European Institute of Technology, Center for Zoonoses, University of Veterinary and Pharmaceutical Sciences, 61242 Brno, Czech Republic; zureklu@vfu.cz; 4 Department of Chemistry and Biochemistry, Mendel University, 61300 Brno, Czech Republic; 5 Arthropod-borne Animal Diseases Research Unit, USDA-ARS, 1515 College Avenue, Manhattan, KS 66502, USA

**Keywords:** house fly, bacteria, dairy farm, urban, agriculture, antimicrobial resistance, AMR, antibiotic, multidrug resistance, MDR

## Abstract

Adult house flies frequent microbe-rich sites such as urban dumpsters and animal facilities, and encounter and ingest bacteria during feeding and reproductive activities. Due to unique nutritional and reproductive needs, male and female flies demonstrate different interactions with microbe-rich substrates and therefore dissemination potential. We investigated culturable aerobic bacteria and coliform abundance in male and female flies (n = 107) collected from urban (restaurant dumpsters) and agricultural (dairy farm) sites. Whole-fly homogenate was aerobically cultured and enumerated on nonselective (tryptic soy agar; culturable bacteria) and selective (violet-red bile agar, VRBA; coliforms) media. Unique morphotypes from VRBA cultures of agricultural flies were identified and tested for susceptibility to 14 antimicrobials. Female flies harbored more bacteria than males and there was a sex by site interaction with sex effects on bacterial abundance at the urban site. Coliform abundance did not differ by sex, site or sex within site. Both male and female flies carried antimicrobial-resistant (AMR) bacteria: 36/38 isolates (95%) were resistant to ≥1 antimicrobial, 33/38 were multidrug-resistant (≥2), and 24/38 isolates were resistant to ≥4 antimicrobials. Our results emphasize the role of house flies in harboring bacteria including AMR strains that pose a risk to human and animal health.

## 1. Introduction

Due to the nutritional requirements of their larvae [1,2], house flies (*Musca domestica* L.) have life-long associations with microbe-rich substrates such as animal excrement, soiled bedding, spoiled food, and refuse [3,4,5]. These substrates exist in both urban and agricultural areas in places such as trash receptacles and livestock rearing/feeding operations, respectively. Adult flies acquire and harbor bacteria from these sites on their external surfaces and in their digestive tract [6]. Surveys have identified over 200 different species of microbes associated with wild-caught house flies [6] and a single house fly can harbor up to 100 different potentially pathogenic microbes [7]. Wild house flies harbor both pathogenic and nonpathogenic bacteria [6,8,9], including those that are antimicrobial resistant (AMR) [10,11]. Because flies associate with a wide variety of microbe rich sites across the landscape, it is not surprising that they harbor different microbial communities associated with the sites with which they interact [12].

While coprophagy is not obligate, both male and female house flies are attracted to and opportunistically ingest nutrient- and microbe-rich substrates such as manure [3]. However, distinct nutritional and reproductive needs between male and female flies likely underlie sex-specific interactions with microbe-rich substrates. Although both males and females can subsist on primarily sugar diets, female house flies are anautogenous, requiring a proteinaceous meal for successful egg development [7,13]. Females can acquire dietary protein by consuming manure or animal secretions and excretions. In addition, female flies are also driven to seek out potential oviposition sites such as animal manure [14], with preference being for microbe-rich substrates to ensure proper nutrition for successful larval development [1,2]. Thus, it follows that male and female flies would have distinct interactions with the substrates with which they interact, which subsequently may be reflected in differences in bacterial carriage. 

In this study we used a culture-based approach to assess the role of house fly sex (male, female) and site (urban, agricultural) on the abundance of both aerobic bacteria and coliforms. In addition, we selected a subset of coliforms from flies from the agricultural site and tested their antimicrobial susceptibility. We hypothesized that female flies would harbor more bacteria than males due to their greater interactions with microbe-rich oviposition substrates, and that coliform abundance would be higher in agricultural flies than urban flies due to open access to large amounts of animal manure.

## 2. Materials and Methods

### 2.1. House Fly Collection

Adult male and female house flies were collected from two environments (sites) in Manhattan, KS: urban (downtown business area, near restaurant dumpsters) and agricultural (dairy cattle facility, Kansas State University,) on three collection dates in 2017 (June 13, July 13 and July 31). In total, 107 house flies with a minimum of 5 flies in each sex at each collection date were sampled (Table 1). House flies were captured by a sweep net, individuals were separated on site into sterile Eppendorf tubes using clean forceps, and tubes of flies were transported to the laboratory on wet ice to minimize activity. Clean sweep nets were used between sites to eliminate cross contamination.

### 2.2. Fly Processing for Bacterial Culture and Enumeration

In the laboratory, body mass (mg) of each male and female house fly was measured (Adam Equipment, Oxford, CT, USA) then individual flies homogenized in 500 µl sterile phosphate-buffered saline (PBS; Fisher Scientific, Pittsburg, PA, USA) using a sterile pestle. The volume of homogenate was adjusted to 1.0 mL with sterile PBS and homogenate was 10-fold serially diluted. A number of dilutions from each fly were plated in duplicate on tryptic soy agar (TSA, BD Difco, Franklin Lakes, NJ, USA) for aerobic bacteria enumeration and violet red bile agar (VRBA, Thermo Scientific Remel, Waltham, PA, USA) for coliform enumeration. Culture plates were incubated at 37 °C for 24 to 48 h. Enumeration of bacterial and coliform colony-forming units (CFUs) that grew on respective dilutions of each media were performed, with all CFUs being counted on TSA and only bright pink CFUs (i.e., coliforms) being counted on VRBA.

### 2.3. Selection and Identification of Morphotypes from VRBA Cultures of Dairy Farm Flies

Increasing evidence suggests that agricultural animals are reservoirs of antimicrobial resistance genes and bacteria [15]. Further, due to their dispersal behavior, house flies can disseminate bacteria from farms to human habitats [11]. Therefore, bacterial colonies with similar gross morphologies on VRBA plates from dairy farm fly cultures were grouped and a representative of a group was selected and sub-cultured on TSA. In total, 38 unique colony morphotypes (hereafter “isolates” representing both coliforms and non-coliforms) were selected and taxonomic affiliation of each isolate was determined using API 20E test kit following manufacturer’s protocol (BioMerieux Inc, Cambridge, MA, USA).

### 2.4. Antimicrobial Susceptibility Testing

To examine the antimicrobial susceptibility and/or resistance of individual isolates, the minimum inhibitory concentration (MIC) was determined. The microplate dilution method [16] was implemented to determine the MIC using commercially available Sensititre™ NARMS Gram Negative Plate (ThermoFisher Scientific, Waltham, MA, USA). The plate consisted of different concentrations of cefoxitin, azithromycin, chloramphenicol, tetracycline, ceftriaxone, amoxicillin/clavulanic acid (2:1 ratio), ciprofloxacin, gentamicin, nalidixic, ceftiofur, sulfisoxazole, trimethoprim/sulfamethoxazole, ampicillin, streptomycin. Detailed information of antimicrobial concentrations is available from the manufacturer’s website (https://assets.thermofisher.com/TFS-Assets/MBD/Specification-Sheets/Sensititre-Plate-Layout-CMV3AGNF.pdf; accessed on 27 June 2020). The assay was performed following the manufacturer’s instructions except plates were incubated at 35 °C for 20 h. Bacterial growth was recorded by measuring the absorbance at 630nm using a microplate reader spectrophotometer (Synergy^TM^ HT, BioTek Instruments Inc., Winooski, VT, USA). The MIC was determined by recording the lowest concentration of antimicrobial that inhibited the bacterial growth. Also, if there was no growth of bacteria in any tested concentration of antimicrobial except in the positive control, MIC was recorded as less than or equal to the lowest concentration of the tested antimicrobial. Similarly, if there was no growth inhibition in any tested concentration, MIC was recorded as more than the highest concentration of antimicrobial. Further, antimicrobial susceptibility and resistance was determined by comparing results (MICs) with Clinical and Laboratory Standards Institute (CLSI) M100-ED28 performance and standards for antimicrobial susceptibility testing guidelines for Enterobacteriaceae (http://em100.edaptivedocs.net/dashboard.aspx; accessed on 27 June 2020). 

### 2.5. Statistical Analysis

All data were analyzed in the R statistical platform [17]. The CFU counts were log transformed and a Shapiro-Wilk test was performed to examine if counts and/or residuals of model (see below) were normally distributed (*p* > 0.05). Using the lme4 package [18] we developed a linear mixed-effect model for each response variable (culturable bacterial and coliform CFUs) to evaluate the effect of sex, site, and sex-by-site interaction. We did not include collection date as a fixed effect in the models because it did not have significant effect on both culturable bacterial and coliform CFUs. To account for pseudo-replication caused by repeated sampling of flies in the same sites over time, we included collection date as a random effect in the model. Subsequently, least-square means (lsmeans) were calculated and pairwise comparisons of lsmeans were made to determine the significant differences among sites and sexes for each response variable using lsmeans package [19]. We further evaluated the effect of sex, site, and their interaction on each response variable (culturable bacterial and coliform CFUs) for each sampling time. A subset of data for individual collection date was prepared to examine if the bacterial or coliform abundance in house flies was significantly affected by sex, site and their interaction within each collection date. A general linear model and pairwise comparisons of lsmeans were performed.

A general linear model was used to test the effect of sex, site, and their interaction on fly body mass. To determine if there was a significant variation in fly body mass between sex a pairwise comparison of lsmeans was performed. Finally, general linear models were applied to determine the relationships of body mass of each sex with culturable bacterial or coliform CFUs. All statistical tests with *p* < 0.05 were considered significant.

## 3. Results

### 3.1. Effect of Sex and Site on Culturable Bacterial Abundance in House Flies

**Figure 1:**
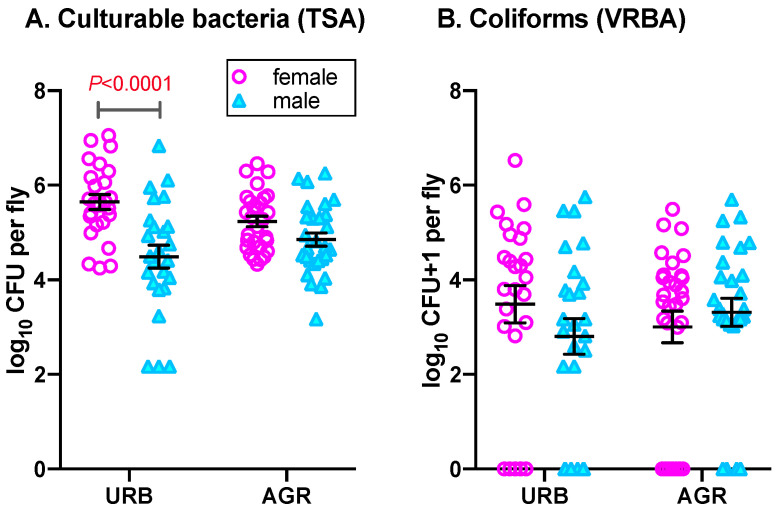
Bacterial abundance in female and male house flies collected from urban (URB) and agricultural (AGR) environments. (**A**) Culturable CFUs isolated on tryptic soy agar (TSA); (**B**) Coliform CFUs isolated on violet red bile agar (VRBA). Mean Log_10_ CFU, standard errors and significant differences in means are shown. Statistical analysis details are in the text.

Irrespective of collection date and fly sex, culturable bacterial CFUs recovered from house flies collected from urban and agricultural sites ranged from 1.49 × 10^2^ to 1.13 × 10^7^ CFU/fly. Overall, there was a statistically significant effect of fly sex on culturable bacterial abundance in house flies (F_(1,103)_ = 21.52, *p* < 0.0001), where female house flies had higher mean culturable bacterial CFUs (1.03 ± 0.59 × 10^6^ CFU/fly) than that of males (3.45 ± 1.37 × 10^5^ CFU/fly; t = 4.64, df =101, *p* < 0.0001). A similar pattern was observed in both urban and agricultural environments. For instance, in the urban environment, a significant difference in mean bacterial CFU was observed between females (1.17 ± 0.59 × 10^6^ CFU/fly) and males (4.43 ± 1.28 × 10^5^ CFU/fly; t = 4.77, df =101, *p* < 0.0001; Figure 1A) whereas in the agricultural environment, the mean CFU abundances in females (4.43 ± 1.28 × 10^5^ CFU/fly) was greater than that of males but not statistically significant (males: 2.56 ± 0.86 × 10^5^ CFU/fly; t = 1.68, df =101, *p* = 0.095; Figure 1A). The overall effect of site on bacterial abundance in adult house flies was not significant (F_(1,103)_ = 0.03, *p* = 0.870), and pairwise comparisons of lsmeans showed that site did not affect culturable bacterial CFUs in either fly sex (male: t = −1.53, *p* = 0.130, female: t = 1.77, *p* = 0.080). However, the sex and site interaction significantly affected bacterial abundance in flies (F_(1,103)_ = 5.45, *p* = 0.021). 

Within a collection date, the number of bacterial CFUs in house flies was highly variable (Table 1). Irrespective of sites, there was a significant effect of sex on bacterial abundance in house flies collected on 13 and 31 July (F_(1,37)_ = 11.21, *p* = 0.002; F_(1,37)_ = 10.24, *p* = 0.003, respectively) but not on 13 June (F_(1,21)_ = 0.497, P = 0.489). Bacterial abundance was significantly higher in females collected on 13 and 31 July compared to males (Table 1; t = 3.47, df = 37, *p* = 0.001; t = 3.21, df = 37, *p* = 0.003, respectively) but non significantly higher in females collected on 13 June (t = 0.67, df = 21, *p* = 0.493). Further, pairwise comparisons of lsmeans revealed no effect of site in the culturable bacterial CFUs in each collection date for either sex (13 June - female: t = 0.46, df = 21, *p* = 0.653; male: t = 0.21, df = 21, *p* = 0.834), (13 July - female: t = 1.14, df = 37, *p* = 0.186; male: t = −1.87, df = 37, *p* = 0.069) and (31 June - female: t = 1.21, df = 37, *p* = 0.234; male: t = −0.32, df = 37, *p* = 0.754). There was no significant effect of site on any collection date (13 June: F_(1,21)_ = 0.23, *p* = 0.639; 13 July: F_(1,37)_ = 0.13, *p* = 0.724; 31 July: F_(1,37)_ = 0.38, *p* = 0.540). Also, the effect of sex and site interaction was significant only on 13 July (F_(1,37)_ = 5.18, *p* = 0.029).

### 3.2. Effect of Sex and Site on Coliform Abundance in House Flies

Irrespective of the collection date, fly sex and site, the number of coliform CFUs recovered from house flies ranged from 0–3.38×10^6^ CFUs/fly. Overall, no significant effects of sex on abundance of coliform CFUs in house flies was observed (Figure 1B; sex: F_(1,101.2)_ = 0.28, *p* = 0.596). Similar to the culturable bacterial CFUs, irrespective of site and collection date, females house flies harbored greater mean abundances of coliform CFUs (9.99 ± 6.28 × 10^4^ CFU/fly) than that of males (4.64 ± 1.62 × 10^4^ CFU/fly) but the difference was non-significant (t = 0.52, df = 101, *p* = 0.596). In the urban environment, coliform CFU abundances in females were higher (1.85 ± 1.34 × 10^5^ CFU/fly) than that of males (5.21 ± 2.67 × 10^4^ CFU/fly) but the effect of sex was non-significant (t = 1.35, df = 101, *p* = 0.180). Surprisingly, in the agricultural environment, the mean coliform abundance was slightly less in females (2.63 ± 1.19 × 10^4^ CFU/fly) compared to males (4.13 ± 1.98 × 10^4^ CFU/fly; t = −0.66, df = 101, *p* = 0.509) but again was non-significant. Interestingly, 12 out of 54 female house flies harbored no (0) coliform CFUs and 58.33% (7/12) were from agricultural environment. Similar to females, 10 out of 53 male house flies did not carry coliform bacteria and 60% (6/10) of those were collected from the urban environment. Moreover, irrespective of sex, site and the sex by site interaction had no significant effect on abundance of coliforms in house flies (site: F_(1,101.3)_ = 0.002, *p* = 0.965; interaction: F_(1,101.5)_ = 2.07, *p* = 0.153). Further, pairwise comparison of lsmeans also revealed no significant effect of site within sex (male: t = −1.04, *p* = 0.299; female: t = 0.989, *p* = 0.325) on coliform abundance.

The abundance of coliform CFUs in flies was highly variable within collection date (Table 1). Interestingly, although there were no overall sex effects, within date there was significant effect of sex on coliform abundance in house flies collected on 13 June and 13 July (F_(1,21)_ = 5.60, *p* = 0.028; F_(1,37)_ = 10.53, *p* = 0.002, respectively). On 13 June, the mean abundance of coliforms in females (1.52 ± 1.15 × 10^4^ CFU/fly) was less than males (4.49 ± 2.31 × 10^4^ CFU/fly; t = −2.47, df = 21, *p* = 0.022). Unlike June, on 13 July mean abundance of coliforms in females (2.23 ± 1.59 × 10^5^ CFU/fly) was greater than of males (6.43 ± 3.71 × 10^4^ CFU/fly; t = 3.41, df = 37, *p* = 0.001) but no such difference was observed on 13 July in mean abundance of coliforms in females (2.56 ± 1.54 × 10^4^ CFU/fly) and males (3.01 ± 1.66 × 10^4^ CFU/fly; t = −0.04, df = 37, *p* = 0.973). Also, the effect of sex and site interaction was significant only on 13 July (F_(1,37)_ = 13.56, *p* = 0.0007). Furthermore, on July 13, a comparison of means revealed a significant effect of sex within site (urban: t = 4.85, df = 37, *p* < 0.0001; agriculture: t = −0.20, df = 37, *p* = 0.840) and site within sex (female: t = 1.77, df = 37, *p* = 0.084; male: t = −3.42, df = 37, *p* = 0.002). No significant effect of interactions in other collection dates were observed.

### 3.3. Relationships of House Fly Body Mass, Sex, Site and Bacterial Abundance

Although the mean mass of female flies (20.81 ± 0.65 mg/fly) was significantly greater than that of males (13.13 ± 0.42 mg/fly; F_(1,103)_ = 98.16, *p* < 0.0001; Figure S1A), the relationship between culturable bacteria abundance and mass was not significant within each fly sex (female: F_(1,52)_ = 0.30, *p* = 0.585; male: F_(1,51)_ = 0.407, *p* = 0.526; Figure S1B). Similarly, the relationship between fly body mass and abundance of coliforms was not significant within each fly sex (female: F_(1,52)_ = 0.23, *p* = 0.631; male: F_(1,51)_ = 0.77, *p* = 0.526). Furthermore, there was no significant effect of site (F_(1,103)_ = 0.65, *p* = 0.422) and interaction on fly body mass (F_(1,103)_ = 0.94, *p* = 0.335).

### 3.4. Bacterial Isolates and Antimicrobial Susceptibility

**Figure 2:**
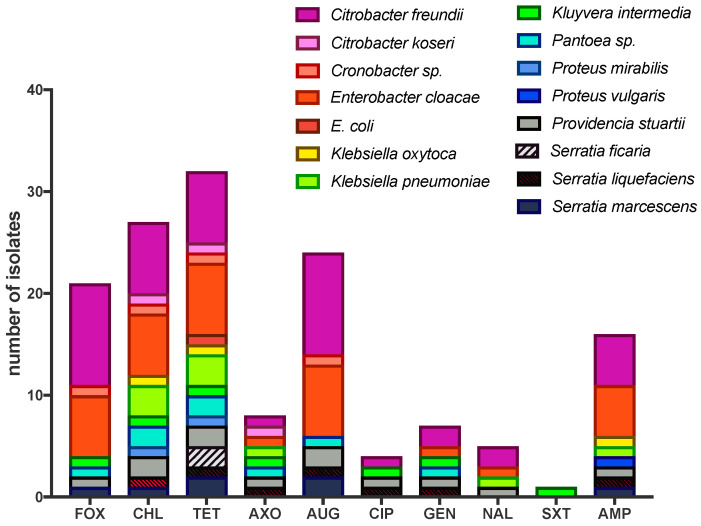
Antimicrobial resistant (AMR) isolates from house flies collected at the agricultural site (dairy farm). Antimicrobial sensitivity testing was performed using the MIC method (details in text) on the Sensitre^®^ Gram negative NARMS plate. Antimicrobial abbreviations: FOX, cefoxitin; CHL, chloramphenicol; TET, tetracycline; AXO, ceftriaxone; AUG, amoxicillin/clavulanic acid (2:1); CIP, ciprofloxacin; GEN, gentamicin; NAL, CIP, Ciprofloxacin, NAL, nalidixic, SXT, trimethoprim-sulfamethoxazole; AMP, ampicillin. MIC values for all isolates and additional antimicrobials are presented in Table S1.

In total, 38 isolates were selected from VRBA cultures of house flies from the agricultural site irrespective of collection date and sex, including both non-coliform and coliform morphotypes: *Citrobacter freundii* (10), *C. koseri/farmeri* (1), *Cronobacter* sp. (1), *Enterobacter cloacae* (7), *Escherichia coli* (1), *Klebsiella oxytoca* (2), *K. pneumoniae* (3), *Kluyvera intermedia* (1), *Pantoea* sp. (2), *Proteus mirabilis* (1), *P. vulgaris* (1), *Providencia rettgeri* (1), *Pr. stuartii* (2), *Serratia ficaria* (2), *S. liquefaciens* (1), *S. marcescens* (2). Of these 38 isolates, 36 (95%) were resistant to at least one of the 14 antimicrobials tested (Figure 2). Isolates showed the greatest resistance to tetracycline (32/38; 84%), chloramphenicol (28/38; 74%), amoxicillin/clavulanic acid (24/38; 63%), cefoxitin (21/38; 55%) and ampicillin (16/38; 42%). All 38 isolates were susceptible to azithromycin and sulfisoxazole. Resistance and susceptibility were not determined for ceftiofur and streptomycin due to lack of CLSI standards, although MICs for these antimicrobials are indicated in Table S1. In regard to bacterial species, 10/10 (100%) *Citrobacter freundii* isolates were resistant to cefoxitin and amoxicillin/clavulanic acid, 7/10 (70%) were resistant to either chloramphenicol and/or tetracycline, and 5/10 (50%) were resistant to ampicillin. *Enterobacter cloacae* also exhibited widespread AMR among isolates: 7/7 (100%) were resistant to augmentin and tetracycline, 6/7 (86%) were resistant to either cefoxitin and/or chloramphenicol, and 4/7 (57%) were resistant to ampicillin. Multidrug resistance (MDR; resistant to ≥2 antimicrobials) was observed in 33/36 AMR isolates (92%). In addition, 24/36 (67%) AMR isolates (or 72% of MDR isolates) were resistant to four or more antibiotics. Three isolates were resistant to seven antimicrobials (*E. cloacae, K. intermedia, S. liquefaciens*) and one isolate was resistant to eight antimicrobials (*C. freundii*). Dual resistance to chloramphenicol and tetracycline was observed in 26 isolates (68% of total isolates, 72% of AMR isolates). Of isolates resistant to three antimicrobials, 17 isolates were resistant to chloramphenicol, tetracycline and augmentin (45% of total, 47% of AMR) and resistance to chloramphenicol, tetracycline and cefoxitin was observed in 15 isolates (39% of total, 42% of AMR). Fourteen isolates were resistant to four antimicrobials (cefoxitin, chloramphenicol, tetracycline and augmentin), comprising 37% of total isolates and 39% of those with AMR; the majority of these MDR isolates were either *C. freundii* (n = 5) or *E. cloacae* (n = 5).

## 4. Discussion

This study examined the effects of fly sex and collection site on the carriage of culturable aerobic bacteria and coliforms by house flies. To our knowledge, this is the first study demonstrating that female house flies carry a greater abundance of culturable bacteria than male house flies both overall and in certain environments. While both male and female adult house flies associate with filth, females likely have a greater propensity to associate with microbe-rich substrates during investigation of potential oviposition sites [14]. Likewise, females have distinct nutritional requirements from males where a proteinaceous meal is required for successful egg production and development [13,20]. Therefore, male and female house flies interact distinctly with substrates in different environments, which may subsequently reflect differences in bacterial carriage.

Within the urban site, female flies harbored a greater abundance of culturable bacteria than males but there was no difference in coliform abundance. Further, there was no significant difference in bacteria and coliform abundance between fly sexes in the agricultural site. During the collections, female house flies were consistently observed interacting with waste residue inside dumpsters at the urban site, and at the agricultural site, flies were closely associated with animals, their excretions, feed, and animal waste. Male flies at the urban site were typically observed resting outside of dumpsters on the lid or side panels and at the agricultural site, males were typically resting on the walls of feed bunks and shelter structures. Thus, although sex-specific behaviors were not quantified in this study, we infer that the nutritional requirements and oviposition interests of females would cause them to have increased contact, inspection and ingestion of either high-protein and/or microbe-rich substrates than males. The lack of significant differences in culturable bacterial and coliform abundance between the sexes at the agricultural site may be attributable to differences in the accessibility and distribution of sources of microbes. In the urban site, apart from the dumpsters there were no apparent alternate sources of microbes at the location where flies were collected. However, a previous study revealed that house flies from cattle farms were found in the urban dumpsters in Kansas and disseminated antibiotic resistant *Enterococcus* to the urban environment [11]. Thus, due to their active dispersal behavior [11,21], flies from nearby agriculture areas could have been captured at urban sites. In contrast, the agricultural site had numerous putative sources of microbes including the animals, their waste, manure in the pens, manure piled on site, and four other nearby livestock facilities (beef cattle, swine, poultry, horses) as well as animal waste storage lagoons. In addition, flies seemed to move freely throughout these facilities, potentially accessing animals and their waste at the different locations.

The mean mass of female flies was greater than males but mass within each sex did not correlate with bacterial and coliform carriage. Therefore, although females carried more culturable bacteria than males, it probably was not due to their size. Rather, females carry more bacteria and have greater body mass, due to more fat body for vitellogenesis [22] and carriage of as many as 150 eggs during each gonotrophic cycle [3]. Whether female flies have a larger digestive tract volume or surface area to carry bacteria was not determined in this study, but the relationship between other sex-specific dimorphisms and bacterial carriage warrant a further study.

Interestingly there were no site effects on either culturable bacterial abundance or coliform abundance. We had predicted that flies, irrespective of sex, would carry more coliforms at the agricultural site than the urban site due to the different access to animal manure, which can serve both as a major source of fecal coliforms [23] and a developmental substrate for house fly larvae [24,25]. In contrast, although statistically insignificant, the mean coliform abundance in flies from the urban site was greater than those from the agricultural site. We do not know the specific sources of coliforms at the urban site, but the dumpsters likely contained discarded food (including uncooked animal products from nearby restaurants) and other human-generated waste which can be a source of coliforms. In addition to human generated waste, coliforms can originate from animal farms which are situated in a < 4 km radius of the urban site. Future studies need to include substrate sampling at sites in order to identify bacterial sources for the flies.

Antimicrobial susceptibility was only measured for a subset of unique morphotypes from VRBA of flies collected from the agricultural site, and therefore offers limited inference potential in regard to the quantitative carriage of AMR or MDR bacteria by house flies. However, our data provide insight into the risk that flies pose in harboring and disseminating AMR and MDR bacteria at an agricultural site. The greatest resistance seen in the isolates was to tetracycline, and of the resistant strains, the majority (about 63%) were coliforms (e.g., *Citrobacter, Enterobacter, Escherichia, Klebsiella*) although some anaerogenic lactose fermenters (e.g., *Kluyvera, Pantoea, Serratia*) and potential pathogens (e.g., *Providencia, Proteus*) were also resistant. Widespread tetracycline AMR in livestock-associated bacteria, including coliforms, has been reported previously especially from poultry and cattle operations, and their waste products such as manure [26,27,28,29]. Tetracyclines surpass all other antimicrobial classes in both frequency and quantity of use as a growth promoter in animal agriculture and are also widely used to treat or prevent a variety of bacterial diseases of livestock and poultry [30]. Further, isolation of tetracycline-resistant bacteria from livestock-associated flies has been previously reported [21,31,32,33,34].

Interestingly, we also observed chloramphenicol resistance in the selected isolates, although this antimicrobial is prohibited from the use in food animals [35]. However, related antimicrobials phenicol, florfenicol, although not labeled for use in lactating dairy cattle, are used for treatment, prophylaxis and metaphylaxis for bovine respiratory disease in beef cattle and treatment of pneumonia in swine [36,37,38,39,40,41]. Of note, the *flo* gene product confers non-enzymatic resistance to both, chloramphenicol and florfenicol [42]. In addition, another chloramphenicol resistance gene, *cmlA*, is linked to genes coding resistance to other antimicrobials used in animal agriculture (e.g., aminoglycosides and sulfonamides) and is transmissible on a plasmid, which suggests a mode of resistance absent of selective pressure by the drug itself [43]. As described above, the dairy facility is near several other livestock facilities, including a swine research facility less than 100 m away and a small beef cattle feedlot about 400 m away. Manure from the feedlot pens is stored on site for periods of time and several waste lagoons are present within the vicinity. Although flies were collected at the dairy farm, they may have visited other animals and manure at the adjacent operations where they could have acquired AMR bacteria. 

We also detected multidrug resistance (MDR) in 92% of the resistant (87% of total) isolates. The most common MDR combination was resistance to chloramphenicol and tetracycline (26 isolates), although many of these isolates were also resistant to amoxicillin/clavulanic acid and/or ampicillin. MDR often indicates linked resistance traits that are co-inherited via integrons, transposons or plasmids [44,45,46,47,48]. The genetic basis of resistance in the isolates from our study was not determined. 

Commensal coliforms may not pose direct threat to human or animal health, but they serve as a source of AMR and MDR genes which can be acquired by cohabiting pathogens through horizontal gene transfer [27,49,50]. House flies pose a double threat in potentially facilitating lateral gene transfer between AMR and susceptible bacteria in the fly gut [32,51,52,53]. Furthermore, flies are highly mobile and transitory, moving bacteria from 20–100 km from the acquisition source to other farms or human habitation [11,54]. In addition, laboratory assays have established that house flies can transmit bacteria via surface contact and excreta, demonstrating their role as potential vectors for a variety of bacteria, including those that are AMR (reviewed in [6,10]). This study therefore adds to the growing evidence implicating flies as major players in the ecology of bacteria, the epidemiology of associated diseases, and the dispersal of AMR genes bacteria may carry.

## 5. Conclusions

Our study demonstrated a sex and sex by site effect on bacterial carriage by house flies. The source of both culturable aerobic bacteria and coliforms at the urban site was likely dumpsters, while at the agricultural site it was presumably animals and their manure. Our results also corroborated those from other studies, by demonstrating that house flies carry AMR and MDR bacteria in areas of livestock agriculture. A thorough assessment of the role flies play in harboring and disseminating bacteria, including AMR and MDR strains, and pathogens, is actively being investigated [8,10,12,55,56,57], risk assessment models (e.g., [58]) can be enhanced by identifying and including variables related to bacterial source, the role of fly sex, environmental niche, fly dispersal patterns and farm management practices. Overall, we provide evidence of sex-specific effects on culturable bacterial load that includes AMR and MDR bacteria. Future behavioral studies are required to determine whether time spent in contact with microbe-rich substrates influence the sex specific differences in bacterial carriage by house flies.

## Figures and Tables

**Table 1:** Abundance of culturable bacteria and coliforms in house flies collected from urban and agricultural sites.

Collection Date	Site	Fly Sex	N	Culturable Bacterial CFU (Mean ± SEM)	Coliform CFU (Mean ± SEM)
13-Jun-17	URB	F	6	1.39 ± 1.08 × 10^6^	2.51 ± 2.50 × 10^4^
	URB	M	5	3.93 ± 2.48 × 10^5^	7.05 ± 5.48 × 10^4^
	AGR	F	7	1.63 ± 0.42 × 10^5^	6.64 ± 4.38 × 10^3^
	AGR	M	7	1.75 ± 0.66× 10^5^	2.67 ± 1.10 × 10^4^
13-Jul-17	URB	F	9	3.26 ± 1.38× 10^6^	4.79 ± 3.65 × 10^5^
	URB	M	10	7.99 ± 6.48 × 10^5^	5.74 ± 5.67 × 10^4^
	AGR	F	12	5.00 ± 2.24 × 10^5^	3.10 ± 1.42 × 10^4^
	AGR	M	10	2.75 ± 1.73 × 10^5^	7.12 ± 5.12 × 10^4^
31-Jul-17	URB	F	10	5.14 ± 1.41 × 10^5^	1.68 ± 0.71 × 10^4^
	URB	M	10	1.14 ± 0.51 × 10^5^	3.74 ± 2.87 × 10^4^
	AGR	F	10	5.72 ± 2.55 × 10^5^	3.44 ± 3.07 × 10^4^
	AGR	M	11	2.91 ± 1.54 × 10^5^	2.34 ± 1.93 × 10^4^

CFU: Colony Forming Units; SEM: Standard Error of the Mean, Sites: URB = urban, AGR = agricultural; Sex: F = female, M = male; N = number of flies.

## Supplementary Materials

The following are available online at https://www.mdpi.com/2075-4450/11/7/401/s1, Figure S1: Mass of male and female house flies and relationship to bacterial carriage, Table S1: Minimum inhibitory concentrations (MICs) of bacterial isolates associated with house flies from the agricultural site.

## References

[1] Schmidtmann E.T., Martin P.A.W. (1992). Relationship between selected bacteria and the growth of immature house flies, *Musca domestica*, in an axenic test system. J. Med. Entomol..

[2] Zurek L., Schal C., Watson D.W. (2000). Diversity and contribution of the intestinal bacterial community to the development of *Musca domestica* (Diptera: Muscidae) larvae. J. Med. Entomol..

[3] West L.S. (1951). The Housefly: Its Natural History, Medical Importance, and Control.

[4] Mullen G.R., Durden L.A. (2009). Medical and Veterinary Entomology.

[5] Moon R.D., Hinton J.L., O’Rourke S.D., Schmidt D.R. (2001). Nutritional value of fresh and composted poultry manure for house fly (Diptera: Muscidae) larvae. J. Econ. Entomol..

[6] Nayduch D., Burrus R.G. (2017). Flourishing in filth: House fly–microbe interactions across life history. Ann. Entomol. Soc. Am..

[7] Greenberg B. (1973). Flies and Disease: II. Biology and Disease Transmission.

[8] Gupta A.K., Nayduch D., Verma P., Shah B., Ghate H.V., Patole M.S., Shouche Y.S. (2012). Phylogenetic characterization of bacteria in the gut of house flies (*M usca domestica* L.). Fems Microbiol. Ecol..

[9] Khamesipour F., Lankarani K.B., Honarvar B., Kwenti T.E. (2018). A systematic review of human pathogens carried by the housefly (*Musca domestica* L.). BMC Public Health.

[10] Onwugamba F.C., Fitzgerald J.R., Rochon K., Guardabassi L., Alabi A., Kühne S., Grobusch M.P., Schaumburg F. (2018). The role of “filth flies” in the spread of antimicrobial resistance. Travel Med. Inf. Dis..

[11] Chakrabarti S., Kambhampati S., Zurek L. (2010). Assessment of house fly dispersal between rural and urban habitats in Kansas, USA. J. Kan. Entomol. Soc..

[12] Park R., Dzialo M.C., Spaepen S., Nsabimana D., Gielens K., Devriese H., Crauwels S., Tito R.Y., Raes J., Lievens B. (2019). Microbial communities of the house fly *Musca domestica* vary with geographical location and habitat. Microbiome.

[13] Glaser R.W. (1923). The effect of food on longevity and reproduction in flies. J. Exp. Zool..

[14] Shah R.M., Azhar F., Shad S.A., Walker W.B., Azeem M., Binyameen M. (2016). Effects of different animal manures on attraction and reproductive behaviors of common house fly, *Musca domestica* L.. Parasitol. Res..

[15] Silbergeld E.K., Graham J., Price L.B. (2008). Industrial food animal production, antimicrobial resistance, and human health. Ann. Rev. Public Health.

[16] Wiegand I., Hilpert K., Hancock R.E. (2008). Agar and broth dilution methods to determine the minimal inhibitory concentration (MIC) of antimicrobial substances. Nat. Protoc..

[17] R Core Team (2013). R: A Language and Environment for Statistical Computing.

[18] Bates D., Maechler M., Bolker B., Walker S. (2015). Fitting linear mixed-effects models using lme4. J. Stat. Soft.

[19] Lenth R., Lenth M.R. (2018). Package “lsmeans”. Am. Stat..

[20] Greenberg B. (1959). House fly nutrition. 1. Quantitative study of the protein and sugar requirements of males and females. J. Cell Comp. Physiol..

[21] Nazari M., Mehrabi T., Hosseini S.M., Alikhani M.Y. (2017). Bacterial contamination of adult house flies (*Musca domestica*) and sensitivity of these bacteria to various antibiotics, captured from Hamadan City, Iran. J. Clin. Diag Res. JCDR.

[22] Adams T.S., Nelson D.R. (1969). Effect of corpus allatum and ovaries on amount of pupal and adult fat body in the housefly, *Musca domestica*. J. Insect Physiol..

[23] Sepehrnia N., Memarianfard L., Moosavi A.A., Bachmann J., Rezanezhad F., Sepehri M. (2018). Retention modes of manure-fecal coliforms in soil under saturated hydraulic condition. J. Environ. Manag..

[24] Larraín P., Salas C. (2008). House fly (*Musca domestica* L.) (Diptera: Muscidae) development in different types of manure. Chil. J. Agric. Res..

[25] Khan H.A.A., Shad S.A., Akram W. (2012). Effect of livestock manures on the fitness of house fly, *Musca domestica* L. (Diptera: Muscidae). Parasitol. Res..

[26] Wang L., Yu Z. (2012). Antimicrobial Resistance Arising from Food-Animal Productions and Its Mitigation.

[27] Shin S.W., Shin M.K., Jung M., Belaynehe K.M., Yoo H.S. (2015). Prevalence of antimicrobial resistance and transfer of tetracycline resistance genes in *Escherichia coli* isolates from beef cattle. Appl. Environ. Microbiol..

[28] Dahshan H., Abd-Elall A.M.M., Megahed A.M., Abd-El-Kader M.A., Nabawy E.E. (2015). Veterinary antibiotic resistance, residues, and ecological risks in environmental samples obtained from poultry farms, Egypt. Environ. Monit. Assess..

[29] Noyes N.R., Yang X., Linke L.M., Magnuson R.J., Cook S.R., Zaheer R., Yang H., Woerner D.R., Geornaras I., McArt J.A. (2016). Characterization of the resistome in manure, soil and wastewater from dairy and beef production systems. Sci. Rep..

[30] Granados-Chinchilla F., Rodríguez C. (2017). Tetracyclines in food and feedingstuffs: From regulation to analytical methods, bacterial resistance, and environmental and health implications. J. Anal. Methods Chem..

[31] Nmorsi O.P.G., Agbozele G., Ukwandu N.C.D. (2007). Some aspects of epidemiology of filth flies: *Musca domestica, Musca domestica vicina, Drosophilia melanogaster* and associated bacteria pathogens in Ekpoma, Nigeria. Vector Borne Zoonotic Dis..

[32] Zurek L., Ghosh A. (2014). Insects represent a link between food animal farms and the urban environment for antibiotic resistance traits. Appl. Environ. Microbiol..

[33] Wei T., Miyanaga K., Tanji Y. (2014). Persistence of antibiotic-resistant and -sensitive *Proteus mirabilis* strains in the digestive tract of the housefly (*Musca*
*domestica*) and green bottle flies (Calliphoridae). Appl. Microbiol. Biotechnol..

[34] Usui M., Shirakawa T., Fukuda A., Tamura Y. (2015). The role of flies in disseminating plasmids with antimicrobial-resistance genes between farms. Microb. Drug Resist..

[35] Food and Drug Administration, U.S. Animal Medicinal Drug Use Clarification Act of 1994. https://www.fda.gov/animal-veterinary/acts-rules-regulations/animal-medicinal-drug-use-clarification-act-1994-amduca.

[36] Ciprián A., Palacios J.M., Quintanar D., Batista L., Colmenares G., Cruz T., Romero A., Schnitzlein W., Mendoza S. (2012). Florfenicol feed supplemented decrease the clinical effects of Mycoplasma hyopneumoniae experimental infection in swine in México. Res. Vet. Sci..

[37] Skogerboe T.L., Rooney K.A., Nutsch R.G., Weigel D.J., Gajewski K., Kilgore W.R. (2005). Comparative efficacy of tulathromycin versus florfenicol and tilmicosin against undifferentiated bovine respiratory disease in feedlot cattle. Vet Ther..

[38] Frank G.H., Briggs R.E., Duff G.C., Loan R.W., Purdy C.W. (2002). Effects of vaccination prior to transit and administration of florfenicol at time of arrival in a feedlot on the health of transported calves and detection of *Mannheimia haemolytica* in nasal secretions. J. Am. Vet. Med. Assoc..

[39] Catry B., Duchateau L., Van de Ven J., Laevens H., Opsomer G., Haesebrouck F., de Kruif A. (2008). Efficacy of metaphylactic florfenicol therapy during natural outbreaks of bovine respiratory disease. J. Vet. Pharm..

[40] Rattanapanadda P., Kuo H.-C., Vickroy T.W., Sung C.-H., Rairat T., Lin T.-L., Yeh S.-Y., Chou C.-C. (2019). In vitro and in vivo synergistic effects of florfenicol and thiamphenicol in combination against swine *Actinobacillus pleuropneumoniae* and *Pasteurella multocida*. Front. Microbiol..

[41] Gonzalez-Martin J.V., Elvira L., Lopez M.C., Villalobos N.P., Lopez-Guerrero E.C., Astiz S. (2011). Reducing antibiotic use: Selective metaphylaxis with florfenicol in commercial feedlots. Livest. Sci..

[42] White D.G., Hudson C., Maurer J.J., Ayers S., Zhao S., Lee M.D., Bolton L., Foley T., Sherwood J. (2000). Characterization of chloramphenicol and florfenicol resistance in *Escherichia coli* associated with bovine diarrhea. J. Clin. Microbiol..

[43] Bischoff K.M., White D.G., Hume M.E., Poole T.L., Nisbet D.J. (2005). The chloramphenicol resistance gene *cmlA* is disseminated on transferable plasmids that confer multiple-drug resistance in swine *Escherichia coli*. Fems Microbiol. Lett..

[44] Mirza S.H., Hart C.A. (1993). Plasmid encoded multi-drug resistance in *Salmonella typhi* from Pakistan. Ann. Trop Med. Parasitol..

[45] Fernández-Alarcón C., Singer R.S., Johnson T.J. (2011). Comparative genomics of multidrug resistance-encoding IncA/C plasmids from commensal and pathogenic *Escherichia coli* from multiple animal sources. PLoS ONE.

[46] Leverstein-van Hall M.A., Blok H.E.M., Donders A.R.T., Paauw A., Fluit A.C., Verhoef J. (2003). Multidrug resistance among Enterobacteriaceae is strongly associated with the presence of integrons and is independent of species or isolate origin. J. Infect. Dis..

[47] Carraro N., Rivard N., Burrus V., Ceccarelli D. (2017). Mobilizable genomic islands, different strategies for the dissemination of multidrug resistance and other adaptive traits. Mob. Gen. Elem..

[48] Meunier D., Jouy E., Lazizzera C., Doublet B., Kobisch M., Cloeckaert A., Madec J.-Y. (2010). Plasmid-borne florfenicol and ceftiofur resistance encoded by the *floR* and *blaCMY-2* genes in *Escherichia coli* isolates from diseased cattle in France. J. Med. Microbiol..

[49] Barza M. (2002). Potential mechanisms of increased disease in humans from antimicrobial resistance in food animals. Clin. Infect. Dis..

[50] Hunter J., Shelley J.C., Walton J.R., Hart C.A., Bennett M. (1992). Apramycin resistance plasmids in *Escherichia coli*: Possible transfer to *Salmonella typhimurium* in calves. Epidemiol. Infect..

[51] Fukuda A., Usui M., Okubo T., Tamura Y. (2016). Horizontal transfer of plasmid-mediated cephalosporin resistance genes in the intestine of houseflies (*Musca domestica*). Microbol. Drug Resist..

[52] Petridis M., Bagdasarian M., Waldor M.K., Walker E. (2006). Horizontal transfer of shiga toxin and antibiotic resistance genes among *Escherichia coli* strains in house fly (Diptera: Muscidae) gut. J. Med. Entomol..

[53] Akhtar M., Hirt H., Zurek L. (2009). Horizontal transfer of the tetracycline resistance gene *tetM* mediated by pCF10 among *Enterococcus faecalis* in the house fly (*Musca domestica* L.) alimentary canal. Microbol. Ecol..

[54] Alam M.J., Zurek L. (2004). Association of *Escherichia coli* O157:H7 with houseflies on a cattle farm. Appl. Environ. Microbiol..

[55] Bahrndorff S., Ruiz-González A., de Jonge N., Nielsen J.L., Skovgård H., Pertoldi C. (2020). Integrated genome-wide investigations of the housefly, a global vector of diseases reveal unique dispersal patterns and bacterial communities across farms. BMC Genom..

[56] Bahrndorff S., de Jonge N., Skovgård H., Nielsen J.L. (2017). Bacterial communities associated with houseflies (*Musca domestica* L.) sampled within and between farms. PLoS ONE.

[57] Rahuma N., Ghenghesh K.S., Ben Aissa R., Elamaari A. (2005). Carriage by the housefly (*Musca domestica*) of multiple-antibiotic-resistant bacteria that are potentially pathogenic to humans, in hospital and other urban environments in Misurata, Libya. Ann. Trop. Med. Parasitol..

[58] Cousins M., Sargeant J.M., Fisman D., Greer A.L. (2019). Modelling the transmission dynamics of *Campylobacter* in Ontario, Canada, assuming house flies, *Musca domestica*, are a mechanical vector of disease transmission. R. Soc. Open Sci..

