# Supplementary material for: Environmental and Sex Effects on Bacterial Carriage by Adult House Flies (Musca domestica L.)
Source: Insects. 2020 Jun 28;11(7):401. doi: 10.3390/insects11070401 (PMC7412185; doi:10.3390/insects11070401)
Supplement: Supplementary file 1 [file insects-11-00401-s001.zip › Table S1.docx]

Table S1: Minimum Inhibitory Concentrations (MICs) of bacterial isolates associated with house flies from the agricultural site.

| Taxon | IsolateID | **FOX** | **AZI** | **CHL** | **TET** | **AXO** | **AUG2** | **CIP** | **GEN** | **NAL** | **XNL** | **FIS** | **SXT** | **AMP** | **STR** |
| --- | --- | --- | --- | --- | --- | --- | --- | --- | --- | --- | --- | --- | --- | --- | --- |
| *Citrobacter freundii* | R1.F2.3 | >32 | 8 | 4 | 32 | 0.25 | 32/16 | 0.015 | 0.5 | 2 | 1 | 64 | <0.12/2.38 | 32 | 4 |
| *Citrobacter freundii* | R1.F2.5 | >32 | 8 | 4 | 4 | 0.25 | 32/16 | 0.015 | 0.5 | 2 | 0.5 | 16 | <0.12/2.38 | 16 | 8 |
| *Citrobacter freundii* | R1.F3.1 | >32 | >16 | >32 | 8 | 0.25 | 32/16 | 0.015 | >16 | 2 | >8 | >256 | 1/19 | >32 | 8 |
| *Citrobacter freundii* | R1.F3.2 | >32 | >16 | 32 | 4 | 0.25 | 32/16 | 0.015 | 1 | 4 | 0.5 | 32 | <0.12/2.38 | >32 | 8 |
| *Citrobacter freundii* | R1.F3.6 | >32 | >16 | >32 | >32 | 0.25 | 32/16 | 0.03 | 1 | 4 | 1 | 32 | 0.25/4.75 | 32 | 4 |
| *Citrobacter freundii* | R3.F3.4 | >32 | >16 | 4 | 32 | 0.25 | 32/16 | 0.03 | 1 | 32 | 4 | 64 | <0.12/2.38 | >32 | 32 |
| *Citrobacter freundii* | R1.F3.11 | >32 | >16 | >32 | >32 | 1 | >32/16 | 0.015 | 1 | 2 | 0.5 | 32 | <0.12/2.38 | 16 | 8 |
| *Citrobacter freundii* | R1.F2.7 | >32 | >16 | >32 | >32 | >64 | >32/16 | >4 | >16 | >32 | 0.5 | 32 | <0.12/2.38 | 2 | 32 |
| *Citrobacter freundii* | R1.F2.12 | >32 | >16 | >32 | >32 | 0.25 | 32/16 | 0.015 | 1 | 2 | 0.5 | 16 | <0.12/2.38 | 8 | 8 |
| *Citrobacter freundii* | R1.F3.16 | >32 | >16 | >32 | >32 | 0.25 | 32/16 | 0.015 | 1 | 8 | 0.5 | 16 | <0.12/2.38 | 4 | <2 |
| *Citrobacter koseri/farmeri* | R2.F9.4 | 16 | >16 | >32 | >32 | 4 | 4/2 | 0.12 | 1 | 4 | 1 | 16 | <0.12/2.38 | 2 | 16 |
| *Cronobacter* | R3.F19.1 | >32 | 16 | >32 | >32 | 0.25 | >32/16 | 0.015 | 1 | 16 | 0.5 | >256 | <0.12/2.38 | >32 | 8 |
| *Enterobacter cloacae* | R1.F2.1 | >32 | 16 | 4 | 32 | 0.25 | 32/16 | 0.015 | 0.5 | 8 | 0.5 | 32 | <0.12/2.38 | 32 | 8 |
| *Enterobacter cloacae* | R2.F9.1 | >32 | >16 | >32 | >32 | 0.25 | >32/16 | 0.015 | 0.5 | 8 | 1 | 32 | <0.12/2.38 | 32 | <2 |
| *Enterobacter cloacae* | R2.F9.3 | >32 | >16 | 32 | 32 | 0.25 | 32/16 | 0.015 | 0.5 | 4 | 1 | 32 | <0.12/2.38 | >32 | 4 |
| *Enterobacter cloacae* | R3.F12.3 | >32 | >16 | >32 | >32 | 0.25 | >32/16 | 0.03 | 2 | 4 | 0.5 | 64 | <0.12/2.38 | 4 | 4 |
| *Enterobacter cloacae* | R2.F2.4 | >32 | >16 | >32 | >32 | 1 | >32/16 | 0.015 | 0.5 | 2 | 1 | 64 | <0.12/2.38 | 8 | 4 |
| *Enterobacter cloacae* | R1.F6.13 | >32 | >16 | >32 | >32 | >64 | >32/16 | 0.015 | 0.25 | >32 | 8 | 128 | 0.25/4.75 | >32 | 2 |
| *Enterobacter cloacae* | R3.F1.2 | 4 | >16 | >32 | >32 | 0.25 | >32/16 | 0.03 | 16 | 4 | 0.12 | 32 | <0.12/2.38 | 16 | 64 |
| *Escherichia coli* | R3.F1.5 | 8 | 8 | 2 | >32 | 0.25 | 2/1 | 0.015 | 2 | 2 | 0.5 | 16 | <0.12/2.38 | 2 | 16 |
| *Klebsiella oxytoca* | R1.F1.6 | 2 | 16 | 4 | 4 | 0.25 | 1/0.5 | 0.015 | 0.5 | 8 | 0.5 | 32 | <0.12/2.38 | 16 | 4 |
| *Klebsiella oxytoca* | R1.F12.2 | 2 | >16 | >32 | >32 | 2 | 4/2 | 0.06 | 0.5 | 8 | 1 | 128 | <0.12/2.38 | 32 | 4 |
| *Klebsiella pneumoniae* | R2.F4.1 | 4 | >16 | >32 | >32 | 0.25 | 4/2 | 0.06 | 1 | 4 | 0.5 | 32 | <0.12/2.38 | 32 | 4 |
| *Klebsiella pneumoniae* | R2.F2.1 | 2 | >16 | >32 | >32 | 0.5 | 4/2 | 0.015 | 0.5 | 8 | 0.5 | 16 | <0.12/2.38 | 8 | 8 |
| *Klebsiella pneumoniae* | R2.F4.2 | 2 | >16 | >32 | >32 | 4 | 1/0.5 | 0.5 | 0.25 | >32 | 0.5 | **16** | <0.12/2.38 | 1 | <2.0 |
| *Kluyvera intermedia* | R2.F3.3 | 32 | >16 | >32 | >32 | >64 | 4/2 | >4 | >16 | 16 | 8 | 16 | >4/76 | 8 | 4 |
| *Pantoea* sp. | R2.F15.4 | 0.25 | >16 | >32 | >32 | 0.25 | 4/2 | 0.12 | 1 | 2 | 0.25 | 64 | 0.25/4.75 | <1 | 4 |
| *Pantoea* sp. | R1.F6.9 | 32 | >16 | >32 | >32 | >64 | >32/16 | 0.03 | >16 | 16 | >2 | 64 | 0.5/9.5 | 2 | 32 |
| *Proteus mirabilis* | R3.F12.2 | 4 | >16 | 32 | 32 | 0.25 | 1/0.5 | 0.015 | 1 | 2 | 0.12 | 16 | 0.25/4.75 | <1 | 64 |
| *Proteus vulgaris* | R2.F11.2 | 8 | >16 | 4 | 8 | 0.25 | 4/2 | 0.03 | 0.5 | 2 | 0.25 | 64 | <0.12/2.38 | >32 | 4 |
| *Providencia rettgeri* | R1.F9.1 | 8 | >16 | 16 | 8 | 0.5 | 4/2 | 0.06 | 1 | 4 | 0.25 | 64 | <0.12/2.38 | <1 | 8 |
| *Providencia stuartii* | R3.F10.1 | 4 | >16 | >32 | >32 | 0.25 | >32/16 | 4 | 4 | >32 | 1 | 128 | 0.25/4.75 | 32 | 16 |
| *Providencia stuartii* | R2.F5.2 | >32 | >16 | >32 | >32 | >64 | >32/16 | 0.03 | >16 | 4 | 0.12 | 16 | <0.12/2.38 | 2 | 32 |
| *Serratia ficaria* | R3.F8.2 | 8 | >16 | >32 | >32 | 0.25 | 16/8 | 0.03 | 0.5 | 2 | 0.5 | 32 | <0.12/2.38 | 16 | 4 |
| *Serratia ficaria* | R2.F17.4 | 4 | >16 | 4 | >32 | 0.5 | 4/2 | 0.03 | 0.5 | 2 | 4 | 64 | <0.12/2.38 | 16 | 4 |
| *Serratia liquefacians* | R1.F5.7 | 16 | >16 | >32 | >32 | >64 | >32/16 | >4 | >16 | 1 | 1 | >256 | 0.25/4.75 | >32 | <2 |
| *Serratia marcescens* | R1.F5.4 | 32 | >16 | 32 | >32 | 0.25 | >32/16 | 0.12 | 2 | 1 | 1 | >256 | <0.12/2.38 | 16 | 16 |
| *Serratia marcescens* | R1.F5.5 | 16 | >16 | 8 | 16 | 0.25 | >32/16 | 0.12 | 1 | 1 | 1 | 64 | <0.12/2.38 | 32 | 8 |

Abbreviations for antibiotics: FOX = Cefoxitin, AZI = Azithromycin, CHL = Chloramphenicol, TET = Tetracycline, AXO = Ceftriaxone, AUG2 = Amoxicillin/ clavulanic acid 2:1 ratio, CIP = Ciprofloxacin, GEN = Gentamicin, NAL = Nalidixic, XNL = Ceftiofur, FIS = Sulfisoxazole, SXT = Trimethoprim/ sulfamethoxazole, AMP = Ampicillin, STR = Streptomycin.

Color codes red = resistance, green= susceptible, yellow = intermediate, and white = reference not available, determined based on CLSI M100 S28:2018 Performance Standards for Antibiotic Susceptibility Testing. No CLSI breakpoints were established for XNL and STR so only MIC is shown without color coding.
