# Supplementary material for: Environmental and Sex Effects on Bacterial Carriage by Adult House Flies (Musca domestica L.)
Source: Insects. 2020 Jun 28;11(7):401. doi: 10.3390/insects11070401 (PMC7412185; doi:10.3390/insects11070401)
Supplement: Supplementary file 1 [file insects-11-00401-s001.zip › Figure S1.docx]

**Figure S1:** **Mass of male and female house flies, and relationship to bacterial carriage.** Female flies are larger than males (A.), but the relationship between bacterial abundance and mass within each sex is not significant (B.).
